# Medial quadriceps tendon–femoral ligament reconstruction for recurrent patellofemoral instability: a systematic review

**DOI:** 10.1007/s00590-025-04650-5

**Published:** 2026-01-13

**Authors:** Filippo Migliorini, Luise Schäfer, Raju Vaishya, Jörg Eschweiler, Francesco Simeone, Nicola Maffulli

**Affiliations:** 1 Department of Trauma and Reconstructive Surgery, University Hospital of Halle, Martin Luther University Halle-Wittenberg, Halle (Saale), Germany; 2 Department of Orthopaedic and Trauma Surgery, Academic Hospital of Bolzano (SABES-ASDAA), Bolzano, Italy; 3 Department of Orthopaedic and Trauma Surgery, Eifelklinik St.Brigida, Simmerath, Germany; 4 Department of Orthopaedics and Joint Replacement Surgery, Indraprastha Apollo Hospitals, New Delhi, India; 5 Department of Trauma and Orthopaedic Surgery, Faculty of Medicine and Psychology, Sapienza University of Rome, Rome, Italy; 6 School of Pharmacy and Bioengineering, Keele University Faculty of Medicine, Stoke on Trent, UK; 7 Department of Life Sciences, Health, and Health Professions, Link Campus University, Rome, Italy; 8 Department of Trauma and Reconstructive Surgery, BG Klinikum Bergmannstrost Halle GmbH, Halle (saale), Germany; 9 Centre for Sports and Exercise Medicine, Queen Mary University of London, Barts and the London School of Medicine and Dentistry, Mile End Hospital, London, UK

**Keywords:** Patellar dislocation, Quadriceps tendon, Knee stability, Ligament reconstruction, Adolescent orthopaedics, Surgical outcomes, Rehabilitation

## Abstract

**Introduction:**

Recurrent patellofemoral instability is frequent in adolescents and young adults, often associated with pain, cartilage damage, and functional impairment. While medial patellofemoral ligament (MPFL) reconstruction is considered the gold standard, it has limitations in skeletally immature patients and revision settings. Increasing attention has therefore been given to the reconstruction of medial quadriceps tendon–femoral ligament (MQTFL), a complementary stabiliser of the patella as the procedure avoids patellar bone tunnels and may reduce complications. However, current evidence on its clinical outcomes remains limited and heterogeneous, highlighting the need for systematic evaluation.

**Methods:**

This systematic review was conducted in accordance with the 2020 PRISMA statement. PubMed, Google Scholar, EMABSE, and Web of Science were accessed in September 2025. No time constraints were used for the search. All clinical studies investigating MQTFL reconstruction, either performed in isolation or in combination with MPFL reconstruction, in patients with recurrent patellofemoral instability were considered for eligibility. The methodological quality of the included studies was assessed using the Cochrane ROBINS-I tool for non-randomised studies.

**Results:**

Data from 322 patients were included in the present analysis. The mean age was 19.7 ± 6.33 years, and 67.8% (218 of 322) were female. The Kujala score increased by 34.1 points (95% CI 25.2–43.0; *P* = 0.013). The Lysholm score improved by 29.4 points (95% CI 10.9–47.8; *P* = 0.031). The Tegner activity scale improved by 1.7 (95% CI 0.7–2.7; *P* = 0.006). The IKDC score improved by 31.4 points (95% CI 26.3–36.5; *P* = 0.008). The mean time to return to sport was 5.4 ± 1.3 months (range 4.0–6.5 months). 74% (173 of 234) of patients resumed athletic activity after MQTFL reconstruction. When considering the level of participation, 69% (50 of 72) of patients were able to return to their preinjury level. The overall complication rate was 7% (24 of 322 procedures), including subluxation (4%, 12 of 273) and dislocation (2%, 5 of 275). The rate of revision surgery for persistent symptoms or functional impairment was 2% (5 of 256 procedures).

**Conclusion:**

MQTFL reconstruction is a promising surgical option for selected patients with recurrent patellofemoral instability. Its anatomical and technical advantages, particularly the avoidance of patellar bone tunnels, support its growing interest. While early clinical outcomes are encouraging, the current evidence is limited by methodological heterogeneity and small sample sizes. Further high-quality, comparative studies are needed to clarify its indications, optimise the technique, and confirm long-term efficacy.

## Introduction

 Recurrent patellofemoral instability, a challenging orthopaedic condition, primarily affects adolescents and young adults [1–3]. It is typically characterised by repeated lateral dislocation or subluxation of the patella during knee motion, often accompanied by pain, apprehension, mechanical symptoms, and limitations in daily and sports activities [4–6]. The condition may arise following a single traumatic dislocation that fails to heal with sufficient restoration of continuity and tension of the soft tissue restraint, or in the context of underlying anatomical risk factors such as trochlear dysplasia, patella alta, increased tibial tubercle–trochlear groove distance, or generalised ligamentous laxity [7–9]. The incidence of primary patellar dislocation in adolescents is estimated at 29 to 43 per 100,000 individuals annually, with recurrence rates as high as 70% in skeletally immature patients managed nonoperatively [10, 11]. Recurrent dislocations can lead to cartilage damage, patellofemoral arthritis, and progressive deterioration of joint function, particularly when inadequately addressed [12–15]. Consequently, surgical intervention is often indicated after the failure of conservative treatment or in the presence of high-risk anatomical features [16–20]. Over the past two decades, various surgical techniques have been developed to restore medial patellar stability [21–23]. The reconstruction of the medial patellofemoral ligament (MPFL) has become the gold standard in most clinical settings, supported by extensive biomechanical and clinical evidence [24–27]. However, despite its widespread use, the MPFL has limitations, particularly in patients with open physes, hypoplastic femoral condyles, or undergoing revision surgery [28–30]. Moreover, over-tightening of the MPFL graft or malpositioning of femoral tunnels may result in altered joint kinematics, pain, or medial overconstraint [31–35]. In this context, increasing attention has been directed toward the medial patellofemoral complex (MPFC) as a whole, with particular interest in the medial quadriceps tendon–femoral ligament (MQTFL) as a complementary and functionally relevant stabiliser of the patella [25, 36]. The medial patellofemoral complex comprises the MPFL and the MQTFL, which together provide the primary soft-tissue restraint against lateral patellar displacement in early knee flexion [37, 38]. Within this complex, the MPFL has traditionally been emphasised, but more recently the MQTFL has gained particular interest as a distinct anatomical structure, with its patellar insertion blending into the quadriceps tendon rather than the medial border of the patella [25]. The MQTFL contributes to medial restraint and dynamically supports patellar tracking during knee extension [38, 39]. Anatomical studies have confirmed its consistent presence and described its femoral and quadriceps attachments in detail, laying the groundwork for surgical reconstruction techniques that aim to replicate its physiological function [40, 41]. Reconstruction of the MQTFL has recently emerged as a promising surgical option for treating recurrent patellofemoral instability, particularly in skeletally immature patients or those with challenging anatomical scenarios [42, 43]. The technique preserves the patellar bone stock, reduces the risk of iatrogenic fractures, and allows for a more anatomic graft path with improved isometry. Furthermore, it may offer an alternative in cases where MPFL reconstruction is contraindicated or has previously failed [44].

Despite the growing interest in MQTFL reconstruction, the available evidence remains limited and dispersed across small cohort studies. To date, no comprehensive systematic review has been conducted to assess the clinical outcomes, complication rates, and methodological quality of published studies focused on reconstruction of the MPFC, with particular emphasis on the relatively underexplored MQTFL reconstruction technique. However, essential uncertainties remain regarding whether different MPFC reconstruction strategies, as isolated MQTFL or combined techniques, offer reproducible results, how they interact with adjunctive interventions such as lateral release or tibial tubercle osteotomy, and how outcomes vary across different patient populations. The purpose of this systematic review was therefore to synthesise the available clinical evidence on MQTL reconstruction, either performed in isolation or in combination with MPFL (MPFC), in patients with recurrent patellofemoral instability. We aimed to evaluate patient-reported outcome measures (PROMs), return to sport, and the complication rate.

## Methods

### Eligibility criteria

This systematic review was conducted to evaluate the clinical outcomes and complication rates following MQTFL reconstruction, either performed in isolation or in combination with MPFL reconstruction, in patients with recurrent patellofemoral instability. Eligible studies were original clinical articles published in peer-reviewed journals, written in English, and classified as level of evidence I to III. Only studies reporting quantitative clinical outcomes with a minimum follow-up of six months were included. Studies were excluded if they were case reports, conference abstracts, narrative reviews, technical notes without outcome data, biomechanical or cadaveric studies, or if they focused exclusively on MPFL reconstruction without the use of MQTFL techniques.

### Search strategy

This study was conducted in accordance with the 2020 Preferred Reporting Items for Systematic Reviews and Meta-Analyses (PRISMA) guidelines [45] and followed the methodological recommendations of the Cochrane Handbook for Systematic Reviews of Interventions [46]. The literature search was conducted in June 2025 across three electronic databases: PubMed, Scopus, and Web of Science. The framework used for the search was the following:

- Patients: recurrent patellofemoral instability;
- Comparator: MQTFL/ MPFC reconstruction;
- Outcomes: PROMs and complications;
- Timing: minimum six months of follow-up.

The strings of medical subject headings (MeSH) used for the database search are reported in Table 1. Additional records were identified through manual screening of the reference lists of included articles.

**Table 1:** MeSH used for the database search (WoS: web of Science)

Database	Search String
PubMed	(“medial quadriceps tendon femoral ligament"All Fields OR “MQTFL” OR “medial patellofemoral complex"All Fields OR “MPFC”) AND (“recurrent patellofemoral instability"All Fields OR “patellar dislocation"MeSH Terms OR “patellar instability”) AND (reconstruction OR surgery)
Scopus	TITLE-ABS-KEY(“medial quadriceps tendon femoral ligament” OR “MQTFL” OR “medial patellofemoral complex” OR “MPFC”) AND TITLE-ABS-KEY(“recurrent patellofemoral instability” OR “patellar dislocation”) AND TITLE-ABS-KEY(reconstruction OR surgery)
WoS	TS=(“medial quadriceps tendon femoral ligament” OR “MQTFL” OR “medial patellofemoral complex” OR “MPFC”) AND TS=(“recurrent patellofemoral instability” OR “patellar dislocation”) AND TS=(reconstruction OR surgery)

### Selection and data collection

Two reviewers (* & *) independently screened the titles and abstracts of all records identified through the database search. This initial screening phase aimed to exclude duplicates and studies that did not meet the preliminary eligibility criteria. For all records considered potentially eligible, full-text articles were retrieved and thoroughly assessed. Disagreements regarding inclusion were resolved through open discussion and a consensus-based approach. If a consensus could not be reached, a third reviewer (*) was consulted to reach a final decision. The same two reviewers (* & *) then independently performed data extraction using a standardised and piloted form designed to capture all relevant information. All extracted data were subsequently verified and cross-checked for consistency and accuracy by both reviewers. Any discrepancies were resolved by consensus.

### Data items

Extracted variables included author, year of publication, journal name, study design, sample size, mean age of patients, length of follow-up, graft type used for MQTFL reconstruction, and any associated procedures such as MPFL reconstruction or lateral release. Clinical outcomes were reported using validated patient-reported outcome measures (PROMs), including the Kujala score [47], Tegner Activity Scale [48], Lysholm Knee Scoring Scale [49], and International Knee Documentation Committee (IKDC) [50]. For each score, both baseline and final follow-up means and standard deviations were recorded. The minimally clinically important difference (MCID) for the Lysholm score was 10/100, 9.1/100 for the Kujala, 15/100 for the IKDC, and 0.5/10 for the Tegner score [51–54]. All reported complications were recorded.

### Methodological quality assessment

The risk of bias for non-randomised studies was assessed using the Risk Of Bias In Non-randomized Studies of Interventions (ROBINS-I) tool [55], developed by the Cochrane Bias Methods Group and the Cochrane Non-Randomised Studies Methods Group. This instrument evaluates the internal validity of studies where the intervention is not randomly assigned, comparing each study to a hypothetical ideal randomised trial. It includes seven domains through which bias may be introduced: bias due to confounding, bias in selection of participants into the study, bias in classification of interventions, bias due to deviations from intended interventions, bias due to missing data, bias in measurement of outcomes, and bias in selection of the reported result. Each domain is rated as having a low, moderate, serious, or critical risk of bias, or as having no information when insufficient data are available. The overall risk of bias is determined by the highest level of risk recorded across the domains. The risk of bias was evaluated independently by two reviewers (* & *), and any discrepancies were discussed and resolved.

### Synthesis methods

All statistical analyses were conducted by the first author (*) using Microsoft Excel. All extracted data were synthesised descriptively. Means and standard deviations were aggregated across studies for each PROM. When sufficient data were available, differences between baseline and final follow-up scores were calculated and expressed as mean difference (MD), accompanied by 95% confidence intervals (CI) and p-values. Given the clinical and methodological heterogeneity between studies, including variability in surgical techniques, graft types, and associated procedures, a quantitative meta-analysis was not performed.

## Results

### Study selection

**Fig. 1:**
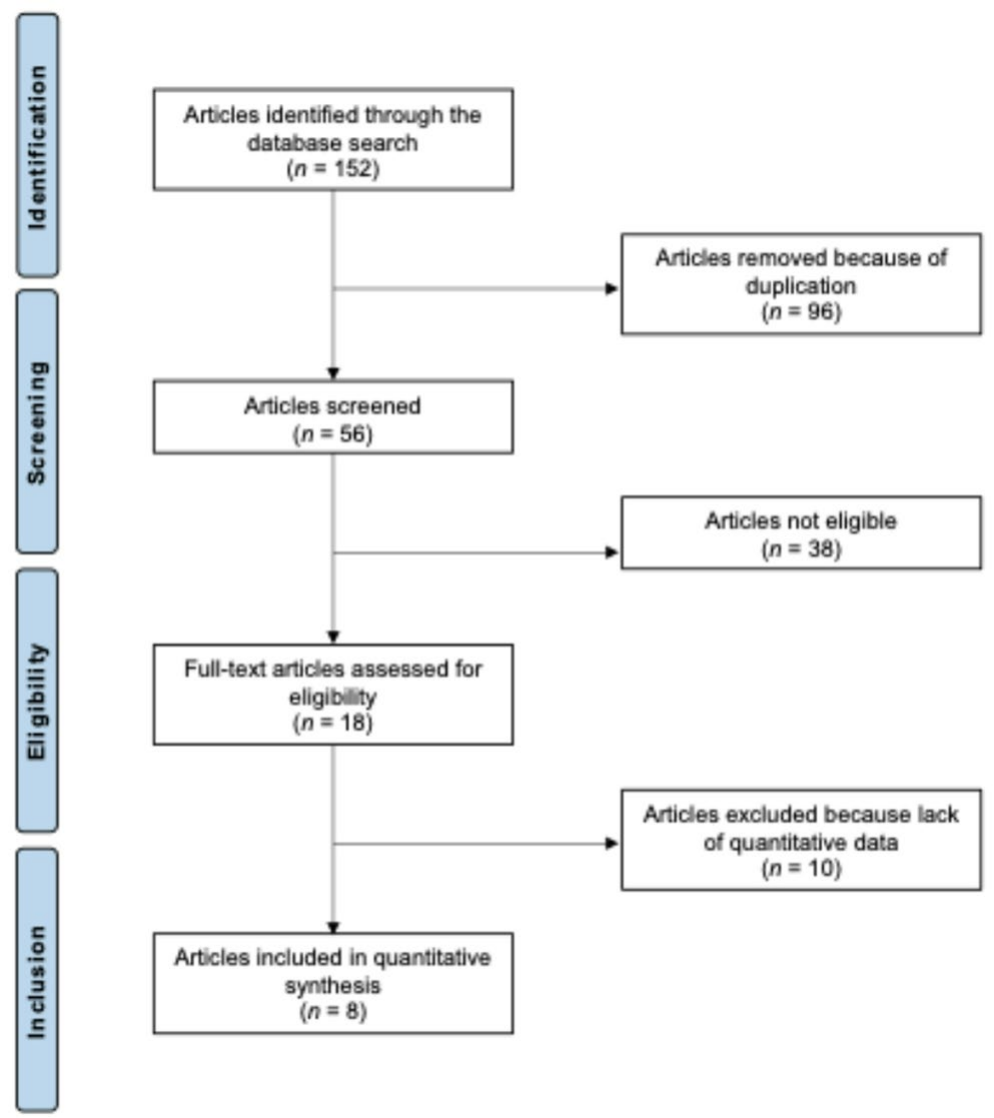
PRISMA flow chart of the literature search

The database search yielded a total of 152 records. After the removal of 96 duplicates, 56 unique studies were available for further assessment. Screening of titles and abstracts led to the exclusion of 38 articles. The main reasons for exclusion were: unsuitable study design (*n* = 9), insufficient level of evidence (*n* = 6), procedures other than MQFTL or MPFC reconstruction (*n* = 12), incomplete description of therapeutic procedures (*n* = 7), and publication in non-eligible languages (*n* = 4). An additional ten studies were subsequently excluded because they did not present quantitative data. Ultimately, eight studies met all inclusion criteria and were retained for analysis. All of them were retrospective investigations with a level of evidence III. The overall selection pathway is illustrated in Fig. 1.

### Risk of bias assessment

The ROBINS-I assessment of methodological quality was applied to all included studies. The domain of confounding was commonly rated as moderate, since although most studies provided relevant baseline characteristics, non-randomised designs and heterogeneity in concomitant procedures limited comparability. Selection of participants was generally assessed as low risk, as explicit inclusion and exclusion criteria were applied, and patients were mainly enrolled consecutively. Likewise, the classification of interventions and deviations from intended interventions were consistently judged to carry a low risk of bias, as surgical techniques were well described and standardised postoperative rehabilitation protocols were followed. Regarding missing data, the majority of studies reported only small proportions of loss to follow-up, resulting in a predominantly low risk rating in this domain. However, some publications raised minor concerns from the incomplete reporting of follow-up rates. The measurement of outcomes was frequently considered to be at moderate risk, since outcomes were largely based on subjective patient-reported measures without blinded assessment. Finally, the selection of reported results was evaluated as low risk, as all prespecified outcomes, including complications, were adequately presented. In summary, while the studies demonstrate methodological strengths such as clear intervention definitions and consistent follow-up, the absence of randomisation and reliance on subjective outcome measures result in an overall low to moderate risk of bias across the body of evidence.

**Fig. 2:**
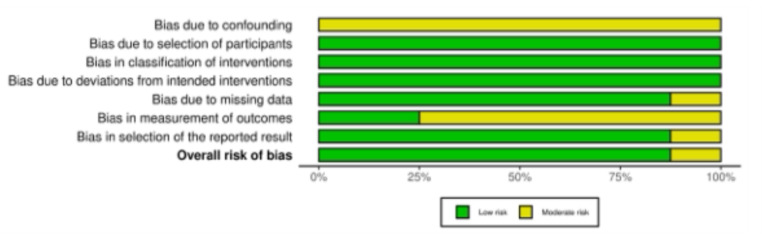
The ROBINS-I of non-RCTs

### Patient demographics

Data from 322 patients were included in the present analysis. The mean age was 19.7 ± 6.33 years, and 67.8% (218 of 322) were female. The general characteristics and patient characteristics are presented in Table 2. Graft selection was heterogeneous and often biased. The semitendinosus autograft was the most frequently used graft, followed by the gracilis autograft. Quadriceps tendon grafts were less frequently used, whereas a few authors used tibialis anterior allografts and peroneus longus. A few articles reported information on previous knee surgery. Most patients had undergone previous ipsilateral knee surgery, including prior MPFL reconstruction, lateral release, chondroplasty, partial lateral meniscectomy, and tibial tubercle osteotomy with revision medial plication and vastus medialis oblique advancement. Pre-injury Tegner activity levels ranged from 6.4 to 6.5, and the mean preoperative tibial tuberosity–trochlear groove (TT-TG) distance ranged from 13.8 to 19.6 mm. Patellar height, assessed using the Insall–Salvati index, ranged from 1.12 to 1.20.

**Table 2:** Generalities of the included studies (LoE: level of evidence; BMI: body mass index; MQTFL-R: medial quadriceps tendon–femoral ligament reconstruction; MPFC-R: medial patellofemoral complex reconstruction)

Author et al., year	Journal name	LoE	Follow up (months)	Treatment	Patients (*n*)	Knee (*n*)	Mean age (y)	Female (*n*)	Mean BMI (kg/m^2^)
Bowman et al., 2021 [56]	*Tech in orthop*	II	29.0	MQTFL-R	12	13	19.0	5	27.0
Hu et al., 2023 [57]	*Arthroscopy*	III	52.1	MPFL-R	70	72	22.3	49	23.5
			48.8	MPFC-R	58	61	22.3	37	22.5
Reikersdorfer et al., 2025 [58]	*Arthroscopy*	III	31.1	MPFC-R	52		12.2	37	
Shankar et al., 2024 [59]	*J ISAKOS*	III	19.7	MQTFL-R	10		28.7	8	26.5
			28.3	MPFL-R	10		29.1	9	26.4
Shi et al., 2023 [60]	*Arthroscopy*	III	47.3	MPFC-R	39	42	22.2	27	21.5
Spang et al., 2019 [61]	*J pediatr Orthop*	III	24.0	MPFC-R	25	27	15.0	15	22.9
Turazza et al., 2025 [62]	*Arthroscopy*	III	28.8	MPFC-R	22	24	13.4	15	
Zein et al., 2024 [63]	*Orthop J Sports Med*	III	40.0	MPFC-R	24	24	12.4	16	

### Results synthesis

The pooled quantitative synthesis showed consistent improvements across all evaluated PROMs (Table 3). The Kujala score increased from 59.3 ± 1.4 preoperatively to 93.4 ± 0.4 at the last follow-up (19.7–52.1 months), representing a 34.1-point improvement (95% CI, 25.2–43.0; *P* = 0.013). The Lysholm score improved by 29.4 points (95% CI 10.9–47.8; *P* = 0.031). The Tegner activity scale showed a significant functional recovery, with a 1.7-point improvement (95% CI, 0.7–2.7; *P* = 0.006). The IKDC score improved by 31.4 points (95% CI 26.3–36.5; *P* = 0.008).

**Table 3:** Results of proms (FU: follow-up; MD: mean difference; CI: confidence interval; IKDC: international knee document Committee)

Outcome	At Baseline	At last FU	MD (95% CI)	*P*
Kujala	59.3 ± 1.4	93.4 ± 0.4	34.1 (25.2–43.0)	0.01
Lysholm	64.5 ± 2.1	93.9 ± 0.1	29.4 (10.9–47.8)	0.03
Tegner activity	3.4 ± 1.8	5.1 ± 0.9	1.7 (0.7–2.7)	0.006
IKDC	55.2 ± 0.2	86.6 ± 0.8	31.4 (26.3–36.5)	0.008

### Return to sport

The mean time to return to sport was 5.4 ± 1.3 months (range 4.0–6.5 months). 74% (173 of 234) of patients resumed athletic activity after MQTFL reconstruction. When considering the level of participation, 69% (50 of 72) of patients were able to return to their preinjury level.

### Complications

The overall complication rate was 7% (24 of 322 procedures), including subluxation (4%, 12 of 273) and dislocation (2%, 5 of 275). The rate of revision surgery for persistent symptoms or functional impairment was 2% (5 of 256 procedures).

## Discussion

According to the main findings of the present systematic review, patients who underwent MQTFL reconstruction reported consistent improvements across all evaluated PROMs beyond their MCIDs. Return to sport was consistently reported across the available studies. The mean time to return to sport was 5.4 ± 1.3 months (range 4.0–6.5 months). 74% (173 of 234) of patients resumed athletic activity after MQTFL reconstruction. When considering the level of participation, 69% (50 of 72) of patients returned to their preinjury level. The overall complication rate was 7% (24 of 322 procedures), including subluxation (4%, 12 of 273) and dislocation (2%, 5 of 275). The rate of revision surgery for persistent symptoms or functional impairment was 2% (5 of 256 procedures). As evidence on isolated MQTFL procedures remains limited, studies addressing MPFC reconstruction, including combined MPFL and MQTFL techniques, were also considered to provide a broader context. Across these approaches, the available studies, although limited in number and heterogeneous in design, consistently demonstrate clinically relevant improvements in PROMs following MQTFL reconstruction, with low complication rates and few reported episodes of redislocation. These findings support the growing interest in this technique as a potentially effective alternative to MPFL reconstruction, particularly in selected patients with challenging anatomical conditions or a history of prior surgical failure.

The MQTFL has garnered increasing attention in recent years as a distinct anatomical structure that contributes to medial patellar stability [64–66]. Anatomical studies have demonstrated that the MQTFL originates from the femoral condyle and inserts into the medial aspect of the quadriceps tendon, blending with the fibres of the vastus medialis obliquus [67, 68]. Its role appears to be both static and dynamic, providing restraint against lateral patellar displacement, especially in early degrees of flexion [38, 39]. In cadaveric investigations, the MQTFL may significantly contribute to patellar stability in conjunction with the MPFL, particularly during dynamic loading conditions [36, 69]. Biomechanical studies have supported these findings by demonstrating that isolated sectioning of the MQTFL leads to increased lateral patellar shift and tilt [38]. Consequently, MQTFL reconstruction has been proposed as a viable alternative to MPFL reconstruction, particularly in skeletally immature patients or in revision surgery, given the absence of patellar tunnel drilling and reduced risk of iatrogenic fracture [38, 70]. While evidence remains limited compared to MPFL reconstruction, preliminary clinical studies suggest that MQTFL reconstruction can result in favourable patient-reported outcomes and low redislocation rates [42, 43]. From a biomechanical standpoint, the MQTFL plays a complementary role to the MPFL in restraining lateral patellar displacement, particularly in the early degrees of knee flexion. Unlike the MPFL, the MQTFL inserts into the quadriceps tendon and therefore contributes to both static and dynamic medial restraint [25, 71]. Reconstruction of this structure offers the theoretical advantage of restoring a more physiological pattern of patellar tracking, thereby avoiding the need for patellar tunnels or hardware fixation. Anatomical studies have confirmed the consistency of its femoral and quadriceps attachments and highlighted its functional relevance in stabilising the patella during active extension [40, 41].

One of the key advantages of MQTFL reconstruction is the avoidance of patellar bone tunnels, thereby reducing the risk of iatrogenic fracture and preserving patellar bone stock [44, 72]. This makes the technique particularly appealing in skeletally immature patients or in revision scenarios where patellar anatomy has been compromised [43]. The graft trajectory more closely follows the anatomical course of the native ligament, allowing for an isometric reconstruction that may enhance functional outcomes [73, 74]. Additionally, fixation into the quadriceps tendon can be less invasive, allowing for more effortless adjustment of graft tension intraoperatively [38, 75]. Nevertheless, several drawbacks must be considered. Fixation in soft tissue, while less invasive, may provide reduced mechanical strength compared with bone tunnels, particularly under repetitive loading. Achieving precise graft tensioning and correct anatomical positioning requires surgical expertise; variations in quadriceps tendon morphology may affect the reproducibility of these procedures. Moreover, in cases involving combined procedures, such as concurrent MPFL reconstruction, the individual contribution of the MQTFL graft to clinical success becomes more challenging to isolate. This could result in an overestimation of its effect or introduce confounding variables when interpreting outcomes.

The overall methodological quality of the included studies was low to moderate. Using the ROBINS-I tool [55], all studies exhibited a moderate or serious risk of bias due to their non-randomised design, lack of blinding, and variability in outcome reporting. There was considerable heterogeneity in patient populations, particularly with respect to skeletal maturity, history of prior surgery, anatomical risk factors (e.g., trochlear dysplasia or increased tibial tuberosity–trochlear groove distance), and chronicity of instability. These variables influence both the surgical indication and the expected outcomes, thereby reducing the comparability of results. Surgical techniques varied markedly between studies. Some authors performed isolated MQTFL reconstruction, while others used combined approaches, including MPFL reconstruction, lateral retinacular release, or tibial tubercle osteotomy. The presence of combined procedures limits the ability to attribute improvements exclusively to the MQTFL graft. Furthermore, the graft types and fixation methods were not standardised. Grafts ranged from gracilis and semitendinosus tendons to quadriceps autografts, while fixation techniques included interference screws, suture anchors, and tunnelless methods. These differences may affect biomechanical integrity, healing, and patient outcomes. There was an inconsistency in rehabilitation protocols and follow-up durations. Some studies initiated early mobilisation, whereas others delayed weight-bearing and range-of-motion exercises. Follow-up ranged from 12 months to over 3 years, complicating long-term comparisons. Additionally, PROMs were not uniformly collected, with some studies using non-validated scores or failing to report both baseline and final values along with their standard deviations. The inconsistent use of the Tegner score and the heterogeneous quality of return-to-sport data further limit functional interpretation. The limited sample sizes in all studies, combined with the absence of power calculations or intention-to-treat analyses, preclude definitive conclusions. Some studies lacked explicit inclusion criteria or did not report how surgical candidacy was determined. These methodological weaknesses reduce both internal and external validity. Future research should prioritise standardised reporting of surgical techniques, clearly defined functional outcomes, and comparative designs that allow differentiation between isolated and combined medial soft-tissue procedures. Such methodological refinement will help clarify the specific contribution of individual reconstruction techniques to patellofemoral stability and long-term patient function.

## Conclusion

MQTFL reconstruction is a promising surgical option for selected patients with recurrent patellofemoral instability. Its anatomical and technical advantages, particularly the avoidance of patellar bone tunnels, support its growing interest. While early clinical outcomes are encouraging, the current evidence is limited by methodological heterogeneity and small sample sizes. Further high-quality, comparative studies are needed to clarify its indications, optimise the technique, and confirm long-term efficacy.

## Data Availability

the datasets generated during and/or analysed during the current study are available throughout the manuscript.
